# By Hook or by Crook? Morphometry, Competition and Cooperation in Rodent Sperm

**DOI:** 10.1371/journal.pone.0000170

**Published:** 2007-01-24

**Authors:** Simone Immler, Harry D.M. Moore, William G. Breed, Tim R. Birkhead

**Affiliations:** 1 Department of Animal and Plant Sciences, University of Sheffield, Sheffield, United Kingdom; 2 Reproductive and Developmental Medicine, University of Sheffield, Sheffield, United Kingdom; 3 Discipline of Anatomical Sciences, School of Medical Sciences, Faculty of Health Sciences, University of Adelaide, South Australia, Australia; University of Oxford, United Kingdom

## Abstract

**Background:**

Sperm design varies enormously across species and sperm competition is thought to be a major factor influencing this variation. However, the functional significance of many sperm traits is still poorly understood. The sperm of most murid rodents are characterised by an apical hook of the sperm head that varies markedly in extent across species. In the European woodmouse *Apodemus sylvaticus* (Muridae), the highly reflected apical hook of sperm is used to form sperm groups, or “trains,” which exhibited increased swimming velocity and thrusting force compared to individual sperm.

**Methodology/Principal Findings:**

Here we use a comparative study of murine rodent sperm and demonstrate that the apical hook and sperm cooperation are likely to be general adaptations to sperm competition in rodents. We found that species with relatively larger testes, and therefore more intense sperm competition, have a longer, more reflected apical sperm hook. In addition, we show that sperm groups also occur in rodents other than the European woodmouse.

**Conclusions:**

Our results suggest that in rodents sperm cooperation is more widespread than assumed so far and highlight the importance of diploid versus haploid selection in the evolution of sperm design and function.

## Introduction

Sperm vary enormously in size and shape across taxa [1]. This variation is largely unexplained, but is thought to be determined by three factors: (i) phylogeny [2]; (ii) mode of fertilisation (internal vs external [3]); and (iii) post-copulatory sexual selection i.e., sperm competition and cryptic female choice [4]. There is strong empirical evidence that sperm competition is a potent driving force in the evolution of sperm traits and is likely to influence the exceptional diversity of sperm design [5]. In primates and rodents for example, sperm trait dimensions including total size and midpiece volume are positively associated with sperm competition [6], [7], [8]. However, our understanding of the functional significance of most sperm traits particularly in the context of sperm competition is still limited.

The sperm of most murine rodents are characterised by a falciform head with an apical hook that varies markedly in size and curvature across species and is absent in a few [9], [10]. The apical hook of rodent sperm is unique among eutherian mammal sperm which typically exhibit a paddle-shaped head. A previous study showed that the highly reflected apical hook of the European woodmouse *Apodemus sylvaticus* (Muridae) sperm was used to form sperm groups or ‘trains’ of up to 50–100 sperm which exhibited increased swimming velocity and thrusting force compared to individual sperm [11]. These sperm ‘trains’ swim faster than individual sperm, especially in viscous media, and hence provide a potential advantage in sperm competition [11]. It was suggested that this form of sperm cooperation is beneficial to some sperm and costly to others [11]. If such cooperation among sperm is advantageous in sperm competition, and if the apical hook determines the formation of sperm groups, we might expect a positive association between both hook shape and curvature and the risk of sperm competition across murine species.

Murine rodents are a species-rich subfamily within the family Muridae and show substantial variation in mating system and hence in the risk of sperm competition across species [12]. Our comparative study of the sperm head morphometry of 37 murine rodent species was designed to test the hypothesis that the shape and curvature of the hook covaried with the risk of sperm competition inferred from relative testis mass [12], [13], [14], [15]. Consistent with this, we found a strong positive association between the shape and curvature of the apical hook and relative testis mass.

## Results

**Figure 1:**
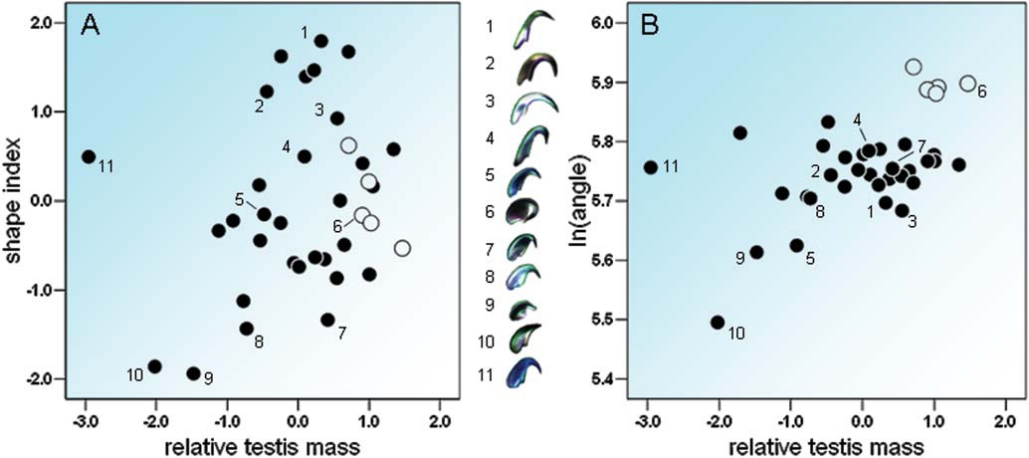
Relationship between hook design and the risk of sperm competition across 37 murine rodent species. Figures are not controlled for phylogeny and residual values of the linear regression between testis mass and body mass were used to obtain relative testis mass. (A) Significant positive relationship between the shape index derived from Principal Component 2 and relative testis mass (testis mass: slope b = 0.59, t = 2.73, p = 0.01, λ = 0.54). (B) Significant positive relationship between the curvature of the apical hook and relative testis mass (slope b = 0.05, t = 4.48, p<0.0001; λ = 0.56). The pictures of sperm heads represent the range of hook design across species: (1) Rattus tuneyi, (2) Mastomys coucha, (3) Leopoldamys sabanus, (4) Niviventer cremoriventer, (5) Bandicota bengalensis, (6) Apodemus argenteus, (7) Maxomys surifer, (8) Acomys cahirinus, (9) Paruromys dominator, (10) Bunomys fratrorum, (11) Notomys alexis. Open circles mark species belonging to the genus Apodemus.

**Figure 2:**
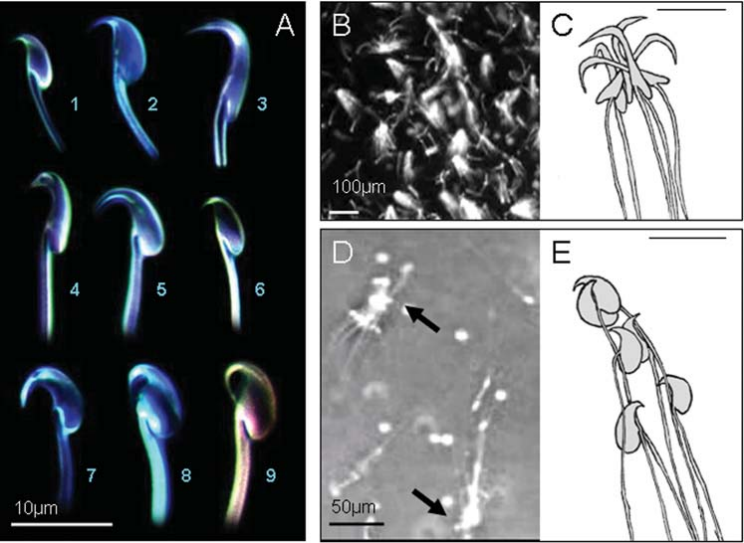
A) Variation in hook design across nine murine rodent species: (1) Bunomys fratrorum, (2) Mus musculus, (3) Rattus norvegicus, (4) Dasymys incomtus, (5) Pseudomys oralis (6) Maxomys surifer, (7) Melomys burtoni, (8) Apodemus sylvaticus, (9) Apodemus speciosus. (B) and (D) frames from videorecording: (B) Approximately 20 sperm groups in the Norway rat R. norvegicus observed in vitro (dark field); (D) Two sperm groups in the house mouse M. musculus observed in vitro (phase-contrast; arrows point at sperm heads). (C) and (E): Drawings showing the attachment of sperm in (C) the Norway rat and (E) the house mouse schematically (scale bar = 10 µm).

We analysed hook shape and hook curvature separately since we were unable to find a single measure that simultaneously encompassed them both (see Methods). Hook shape was investigated by performing an elliptic Fourier analysis [16]. A subsequent Principal Component Analysis (PCA) of the elliptic Fourier coefficients revealed that 56% of the variation in hook shape across species was explained by the first Principal Component (PC1) and 24% by PC2 (see Methods). PC1 explained mainly the difference in hook shape between the genus *Apodemus* and all the other genera, and was not associated with relative testis mass. PC2 explained the extent of the apical hook relative to the size of the sperm head, and we found a significant positive relationship between the length of the apical hook (PC2) and testis mass when controlling for body mass (testis mass: slope *b* = 0.59, *t* = 2.73, *P* = 0.01, effect size *r* = 0.43 (confidence interval CI: 0.12–0.80); body mass: *b* = −0.49, *t* = 1.75, *P* = 0.09, effect size *r* = 0.29 (confidence interval CI: −0.04–0.64), λ = 0.54, n = 37; Figure 1A). Since PC1 explained mainly the difference in shape between the extremely pronounced hook of all *Apodemus* species (Figure 2A) and the hooks of all other genera, we repeated the analysis excluding the five *Apodemus* species. In this analysis, PC1 explained 48% of the variation across species and described the change of the shape of the hook similarly to PC2 in the previous analysis. PC1 was significantly positively associated with testis mass when controlling for body mass (testis mass: slope *b* = 0.63, *t* = 2.77, *P* = 0.01, effect size *r* = 0.46 (confidence interval CI: 0.13–0.86); body mass: *b* = −0.60, *t* = 1.90, *P* = 0.07, effect size *r* = 0.34 (confidence interval CI: −0.01–0.72), λ = 0.58, n = 32).

Hook curvature measured as the angle between the hook and the main axis of the sperm head (see Methods) varied substantially across species (range of angle: 244°–375°; Figure 2A). The relationship between hook curvature and testis mass was significantly positive when controlling for body mass (testis mass: slope *b* = 0.05, *t* = 4.48, *P*<0.0001, effect size *r* = 0.61 (CI: 0.38–1.05); body mass: *b* = −0.07, *t* = 4.40, *P* = 0.0001, *r* = 0.61 (CI: 0.37–1.04), λ = 0.56, n = 37; Figure 1B). The results of the analyses were supported by an intermediate value of the phylogenetic parameter λ indicating that factors other than phylogeny play an important role in the explanation of the observed pattern [17], [18].

To establish whether the apical hook facilitated the formation of sperm groups in murine rodents other than the wood mouse, we conducted an *in vitro* assay, following the methodology in Moore *et al*. [11] in the Norway rat *Rattus norvegicus* (hook angle = 297.5°±1.85 s.e., n = 4; Figure 2A) and the house mouse *Mus musculus* (hook angle = 299.1°±3.86 s.e., n = 7; Figure 2A). Sperm groups occurred in both species; only anecdotal observations had been made previously of sperm aggregations in these species and the motility of sperm groups had not been quantified (HDMM, unpubl. data). In the Norway rat, sperm from the vas deferens and caudal epididymis formed groups of between five and 50 sperm by interlocking at their heads but not at the flagella (Figure 2B+C; see Video S1). In the house mouse, sperm formed groups of three to 30 sperm which attached to each other at both the heads and the flagella (Figure 2D+E). In the house mouse, often several smaller sperm groups attached to each other to form extended groups. In the Norway rat, sperm groups exhibited higher straight-line velocity than individual sperm (laboratory rat: groups: 131 µms^−1^±4 s.e.; individual sperm: 114 µms^−1^±5 s.e., *t* test: *t*_48_ = 2.60, *P* = 0.01; wild rat: groups: 111 µms^−1^±9 s.e., individual sperm: 83 µms^−1^±6 s.e., *t* test: *t*_48_ = 2.59, *P* = 0.01). This was not the case in the house mouse where sperm groups moved more slowly than individual sperm (mouse 1: groups: 112 µms^−1^±4 s.e., individual sperm: 149 µms^−1^±11 s.e., *t*_48_ = 2.86, *P* = 0.006).

## Discussion

Our study revealed a strong positive association between the shape and the curvature of the apical hook of murine sperm and the risk of sperm competition inferred from relative testis mass. Our results are the first evidence that the shape and curvature of the apical hook of rodent sperm heads is influenced by the risk of sperm competition, and that sperm cooperation is likely to be a general pattern in rodents that may have evolved in response to sperm competition.

Sperm competition may be divided into (i) the competition between sperm of rival males (inter-male sperm competition due to diploid selection [19]), and (ii) the competition among the sperm from a single male's ejaculate (intra-male sperm competition due to haploid selection [20]). In the European woodmouse, it has been shown that only those sperm at the tip of a ‘train’ are capable of fertilisation whereas all others undergo the acrosome reaction to separate from each other, rendering them infertile [11]. If sperm cooperation is costly to some sperm and beneficial to others sperm within one ejaculate might compete for the benefiting position. Therefore, if sperm cooperation increases the fertilisation success of a male in sperm competition, diploid selection is expected to drive the evolution of sperm cooperation, whereas haploid selection opposes sperm cooperation if cooperation is costly. The genetic relationship between the sperm of one male is 0.5 which is the same relationship as between full siblings. Therefore, Hamilton's rule for the evolution of cooperation applies [21] and sperm cooperation may still evolve despite haploid genetic influences if the selective pressure (e.g., due to high risk of sperm competition) is intense enough [22]. Sperm cooperation occurs in other taxonomic groups [23]–[25] and in American marsupials, paired sperm perform better in viscous media than individual sperm [24], and in the fishfly, *Parachauliodes japonicus*, swimming velocity increases with increasing number of sperm composing a sperm bundle [25].

The observation of sperm groups in the Norway rat and the house mouse is consistent with our hypothesis that the apical hook plays a role in sperm cooperation in rodents, although in these species the main function of the hook appears to be to maintain the stability of sperm groups rather than the actual attachment of sperm to each other. As in the European woodmouse [11], in the Norway rat and the house mouse sperm attached to each other at the lower ventral region of the apical hook. In the latter two species, as soon as a group was formed the hook appeared to prevent the random detachment of sperm. Sperm separated themselves from the group only by moving rigidly forward. In the European woodmouse, electron-dense adhesive material has been found in the inner curvature of the hook [11] which may facilitate attachment between individual sperm. A similar mechanism might exist in the sperm of the Norway rat and the house mouse. The hypothesis of the stabilising effect of the hook on group formation is supported by the fact that the shape and curvature hook appear to influence the duration for which sperm remain attached to each other: in the Norway rat and in the house mouse, sperm stayed as a group *in vitro* for a maximum of 10 minutes compared to a maximum of 90 minutes in the European woodmouse. In addition, the apical hook in the European woodmouse is flexible and actively moves to lock up with either the hook or flagellum of another sperm which might influence the stability of sperm train formation. No such movement was observed in the Norway rat or house mouse.

The functional significance of sperm groups in rodents is not yet fully understood. An advantage in straight-line velocity does not hold in the house mouse where individual sperm were faster than sperm groups. It is possible that although the sperm groups in the house mouse are slower than individual sperm, they have greater thrusting force. In the European wood mouse, sperm ‘trains’ exhibited increased thrusting force in viscous media [11], which may be advantageous for example to penetrate the cervical mucus in the female reproductive tract. Alternatively, sperm groups may have evolved in response to the gelatinous copulatory plugs left by males during copulation [26]: sperm groups might advance further up the female reproductive tract and therefore avoid being trapped when the plug is formed. A necessary next step therefore is to test the performance of sperm groups of different rodent species including the Norway rat and the house mouse in viscous media and *in situ*.

Other explanations for the evolution of the apical hook of rodent sperm have been proposed but none substantiated. First, the apical hook might facilitate the attachment of sperm to the wall of the female reproductive tract prior to fertilisation [27], although subsequent data have suggested that this hypothesis is unlikely as mouse and rat sperm swim along the epithelium of the female tract by contact with the lateral surface of the sperm head and not the apical hook [28]. Second, the apical hook may physically bind the sperm to the outer zona pellucida surface of the oocyte and/or protect the region of the sperm head that binds to and fuses with the oolemma [29], [30]. A comparative study of three species of conilurine rodents failed to find a relationship between the complexity of the sperm head and the zona thickness [31]. However, further studies are needed to investigate the interaction between sperm and ovum in rodents.

### Conclusion

Sperm cooperation may be the main selective force favouring the evolution of an apical hook which is such a common feature of rodent sperm. The fact that sperm cooperation may be a widespread phenomenon adds new aspects to the mechanisms of postcopulatory sexual selection and sperm competition in particular. Establishing the relative importance of diploid versus haploid selection in the evolution of sperm shape and function should be a major task for future studies.

## Methods

### Analysis of sperm design

Hook shape was assessed using an outline analysis based on an elliptic Fourier analysis [16]. The outline coordinates were obtained using the program tpsUtil Version 1.33 [32]. Eight harmonics yielding 36 coefficients, which described the shape variation across species sufficiently, were calculated from the outline coordinates using the software EFA [33]. Shape was standardised for orientation, location and size of the sperm head, which resulted in the exclusion of three coefficients for further analysis due to invariance. Hence 32 coefficients were included in a Principal Component Analysis (PCA) based on a variance-covariance matrix. In the analysis including the genus *Apodemus*, the first four principal components (PC) explained 56%, 24%, 11% and 6% respectively of the shape variation across species. Multiple regression analyses in a phylogenetic framework [17], [18] as described below were performed on PC1 and PC2 (which together explained 80% of the variation in hook shape). In the analysis excluding the genus *Apodemus*, the first four PCs explained 48%, 34%, 9% and 6% respectively.

Curvature of the apical hook was assessed by measuring the outer angle between the main axis laid through the sperm head and the tangent laid through the most apical tip of the ventral curve of the hook. We measured the curvature of the apical hook of five sperm of one male per species. The repeatability [34] of the hook curvature within males was intermediate to high (ranging from *r* = 0.49, *F* = 5.71_3,16_, *P* = 0.007, n_0_ = 5 in *Dasymys incomtus* to *r* = 0.87, *F* = 34.11_4,20_, *P*<0.0001, n_0_ = 5 in *Mastomys natalensis*). Within species repeatability calculated for five species was high (*r* = 0.90, *F* = 42.20_4,19_, *P*<0.0001, n_0_ = 4.77). Multiple regression analyses in a phylogenetic framework were performed on hook angle.

The analyses of hook shape and curvature were all performed on non-activated sperm. In *Apodemus*, the shape of activated sperm changes as the hook opens and the angle of attachment is around 360° which is still greater than in all other species [11].

To establish the extent to which hook shape and hook curvature were independent we tested whether these traits covaried across all species. Since a positive relationship existed both between (i) hook shape described by PC1 and hook curvature (*r* = 0.42, *t* = 3.00, *P* = 0.005), and (ii) hook shape described by PC2 and hook curvature (*r* = 0.55, *t* = 4.05, *P* = 0.0002), shape and curvature represent two different aspects of sperm design, as is clear from the different positions of *Rattus* and *Apodemus* in the two analyses (compare Figure 1A and Figure 1B in the published text). However, after excluding the genus *Apodemus*, only the relationship between shape described by PC1 and hook curvature was significant (*r* = 0.58, *t* = 4.08, *P* = 0.0003) and therefore a separate analysis of the relationship between hook curvature and relative testis mass was redundant.

Information on testis mass and body mass was obtained from the literature (Table S1).

### Statistical analysis

To account for statistical independence of data points due to shared ancestry we used a generalised least squares approach (GLS) in a phylogenetic framework [17], [18]. Multiple regressions were performed based on maximum-likelihood models (ML) which control for phylogeny by referring to an internal matrix of expected covariances among species based on their degree of shared ancestry. Both testis mass and body mass were included into the model as independent variables to control for the allometry between testis mass and body mass [13]. A phylogenetic tree was constructed from published sources (Figure S1). We assumed a punctuational model of evolution and set branch length to 1. The phylogenetic dependence parameter λ was estimated. The maximum likelihood value of λ was compared against one and zero. Effect size *r* and the confidence intervals were calculated to estimate the strength of the observed pattern independent of the sample size [35].

### *In vitro* assay for sperm groups

Males of two species with intermediate hook curvature were used for the *in vitro* assay of sperm train formation: the Norway rat *Rattus norvegicus* and the house mouse *Mus musculus*. Two captive bred and two wild caught male Norway rats and two male laboratory house mice in breeding condition were killed and dissected immediately and sperm from the caudal end of the epididymis were released into *in vitro* fertilisation medium for laboratory rats and mice [36] at 37°C. For one laboratory rat, one wild rat and one laboratory mouse we video registered the sperm groups to assess straight line velocity by measuring the distance covered and the duration to cover the distance for sperm groups and individual sperm.

## Supporting Information

Table S1 Information on testis mass (TM), body mass (BM) and the hook angle of 37 murine rodent species.
(0.08 MB DOC)

Figure S1 Phylogeny of 37 murine rodent species used for statistical analyses.
(0.03 MB DOC)

Video S1 In vitro videorecording of sperm groups in the Norway rat.
(1.85 MB AVI)
