# Supplementary material for: By Hook or by Crook? Morphometry, Competition and Cooperation in Rodent Sperm
Source: PLoS One. 2007 Jan 24;2(1):e170. doi: 10.1371/journal.pone.0000170 (PMC1764683; doi:10.1371/journal.pone.0000170)
Supplement: Table S1 — Information on testis mass (TM), body mass (BM) and the hook angle of 37 murine rodent species. (0.08 MB DOC) [file pone.0000170.s001.doc]

**Table S1:** Information on testis mass (TM), body mass (BM) and the hook angle of 37 murine rodent species.

| **Species** | **TM[g]** | **BM[g]** | **angle** |
| --- | --- | --- | --- |
| *Dasymys incomtus* | 3.048 | 242 | 306.12 |
| *Aethomys namaquensis* | 1.476 | 72 | 310.19 |
| *Mastomys natalensis* | 0.972 | 59 | 312.42 |
| *Mastomys coucha* | 0.741 | 70 | 312.21 |
| *Mus musculus* | 0.119 | 15 | 300.99 |
| *Apodemus agrarius* | 1.232 | 35 | 361.95 |
| *Apodemus speciosus* | 1.233 | 46 | 374.63 |
| *Apodemus semotus* | 1.378 | 28 | 364.31 |
| *Apodemus argenteus* | 0.651 | 21 | 360.57 |
| *Apodemus sylvaticus* | 0.788 | 23 | 358.24 |
| *Pseudomys australis* | 2 | 59 | 322.98 |
| *Pseudomys desertor* | 0.558 | 39 | 314.98 |
| *Pseudomys fumeus* | 2.426 | 71 | 319.53 |
| *Mastacomys fuscus* | 3.725 | 117 | 319.59 |
| *Pseudomys nanus* | 1.9 | 74 | 314.45 |
| *Pseudomys gracilicaudatus* | 1.084 | 105 | 341.43 |
| *Notomys alexis* | 0.041 | 30 | 316.22 |
| *Notomys cervinus* | 0.224 | 37 | 302.68 |
| *Conilurus penicillatus* | 4.388 | 184 | 311.72 |
| *Mesembriomys gouldii* | 5.498 | 545 | 328.39 |
| *Zyzomys argurus* | 0.434 | 45 | 327.98 |
| *Melomys burtoni* | 1.91 | 78 | 328.77 |
| *Hydromys chrysogaster* | 11.765 | 745 | 323.33 |
| *Leggadina forresti* | 0.545 | 30 | 326.1 |
| *Rattus colletti* | 1.938 | 154 | 321.61 |
| *Rattus tunneyi* | 4.865 | 243 | 297.94 |
| *Rattus lutreolus* | 4.434 | 163 | 308.14 |
| *Rattus norvegicus* | 4 | 289 | 298.424 |
| *Bandicota bengalensis* | 2.121 | 289 | 277.27 |
| *Paruromys dominator* | 1.601 | 342 | 274.14 |
| *Sundamys muelleri* | 7.44 | 400 | 307.01 |
| *Bunomys fratrorum* | 0.407 | 137 | 243.56 |
| *Niviventer cremoriventer* | 1.222 | 75 | 325.25 |
| *Leopoldamys sabanus* | 9.035 | 373 | 294.04 |
| *Maxomys bartelsii* | 4.205 | 93 | 317.67 |
| *Maxomys surifer* | 3.328 | 155 | 315.62 |
| *Acomys cahirinus* | 0.224 | 27 | 300.15 |

**References for information on testis mass and body mass:**

Breed, W. & Taylor, J. 2000. Body mass, testes mass, and sperm size in murine rodents. *J. Mammal.* **81,** 758-768.

Gage, M. J. G. & Freckleton, R. P. 2003. Relative testis size and sperm morphometry across mammals: no evidence for an association between sperm competition and sperm length. *Proc. R. Soc. Lond. B* **270**: 625-672.
