# Supplementary material for: By Hook or by Crook? Morphometry, Competition and Cooperation in Rodent Sperm
Source: PLoS One. 2007 Jan 24;2(1):e170. doi: 10.1371/journal.pone.0000170 (PMC1764683; doi:10.1371/journal.pone.0000170)
Supplement: Figure S1 — Phylogeny of 37 murine rodent species used for statistical analyses. (0.03 MB DOC) [file pone.0000170.s002.doc]

**Figure S1:** Phylogeny of 37 murine rodent species used for statistical analyses.

**References for phylogeny:**

Baverstock, P. R., Watts, C. H. S., Adams, M. & Cole, S. R. 1981. Genetical relationships among Australian rodents (Muridae). *Australian Journal of Zoology*, **29,** 289-303.

Jansa, S. A. & Weksler, M. 2004. Phylogeny of muroid rodents: relationships within and among major lineages as determined by IRBP gene sequences. *Molecular Phylogenetics and Evolution*, **31,** 256-276.

Menzies, J. I. 1996. A systematic revision of *Melomys* (Rodentia: Muridae) of New Guinea. *Australian Journal of Zoology*, **44,** 367-426.

Verneau, O., Catzeflis, F. & Furano, A. V. 1997. Determination of the evolutionary relationships in *Rattus* sensu lato (Rodentia: Muridae) using L1 (LINE-1) amplification events. *Journal of Molecular Evolution*, **45,** 424-436.

Watts, C. H. S., Baverstock, P. R., Birrell, J. & Krieg, M. 1992. Phylogeny of the Australian rodents (Muridae): a molecular approach using microcomplement fixation of albumin. *Australian Journal of Zoology*, **40,** 81-90.
